# Effect of Tactile Imitation Guidance on Imitation and Emotional Availability. A Case Report of a Mother and Her Child With Congenital Deafblindness

**DOI:** 10.3389/fpsyg.2020.540355

**Published:** 2020-10-02

**Authors:** Sini Peltokorpi, Marlene Daelman, Saara Salo, Minna Laakso

**Affiliations:** ^1^Department of Psychology and Speech-Language Pathology, University of Turku, Turku, Finland; ^2^Pediatric Research Center, New Children’s Hospital, University of Helsinki and Helsinki University Hospital, Biomedicum Helsinki, Helsinki, Finland; ^3^Member of Groningen Study Group on Congenital Deafblindness and Diversity in Communication, Brugge, Belgium; ^4^Faculty of Educational Sciences, University of Helsinki, Helsinki, Finland; ^5^Department of Psychology and Logopedics, University of Helsinki, Helsinki, Finland

**Keywords:** Trisomy 13, imitation, emotional availability, dual sensory loss, congenital deafblindness, tactile modality, guidance for parents

## Abstract

Interaction between parents and children with congenital deafblindness (CDB) is easily hampered due to dual sensory loss. This case report examines imitation and emotional availability in interaction between a mother and her 3-year-old child with CDB first in unguided play and then in three play sessions with tactile imitation guidance. The video recorded play sessions were analyzed for frequency, length, and modality of imitation. Emotional Availability Scales were used to code the emotional quality of interaction. The results showed that before the guidance the mother imitated the child mainly vocally. After the guidance, the use of tactility in imitations increased. Imitation exchanges lasted longest in the last session. The emotional availability between the mother and the child was higher after the guidance. Further research is needed to confirm the positive outcomes of this case study.

## Introduction

“Deafblindness is a combined vision and hearing impairment of such severity that it is hard for the impaired senses to compensate for each other. Thus, deafblindness is a distinct disability. To help compensate for the combined vision and hearing impairment, especially the tactile sense becomes important” (Nordens välfärdscenter, 2016). Children with congenital deafblindness (CDB) comprise a heterogeneous group, and they may also have additional cognitive or motor disabilities (Dammeyer, 2012). However, many children with CDB would gain from interventions that allow them to compensate for their vision and hearing loss by providing them access to information and experiences through the tactile modality. This type of sensory compensation may be due to the role of experience in the development of cross-sensory integration and association (Guellaï et al., 2019). Thus, children with CDB can benefit from tactile interactions, in that both symbolic (e.g., manual signs) and non-symbolic communication (e.g., mutual attention) could be modified in the tactile modality (Miles, 2003; Chen and Downing, 2006; Dammeyer et al., 2015; Nafstad and Rødbroe, 2015; McLinden and McCall, 2016).

Characteristically, many children with CDB have limited skills in spoken and sign languages and express themselves through vocalizations, gestures, tactile, or bodily means (Bruce, 2005; Dammeyer and Ask Larsen, 2016). Parents may find it difficult to read the affective states (Fraiberg, 1979) or interpret the expressions of a child with CDB (Daelman et al., 1993; Janssen et al., 2010). Besides, it may be hard for parents to respond in a perceivable manner to their child with CDB, and different strategies have been suggested for overcoming the difficulties (see Hart, 2010; Janssen et al., 2010; Martens et al., 2014b). Sometimes parents can intuitively identify the appropriate strategies in interaction, but often they need assistance. The use of tactile modality in interaction can be particularly difficult to put into practice, because it is not typical for sighted-hearing parents to use touch systematically for communicative purposes, suggesting the relevance of including touch in guidance for parents (Dammeyer, 2011).

It has been suggested that there is a relationship between challenging behavior and communication difficulties. Poor communication with others is related to emotional and behavioral problems in children with developmental disabilities (Sigafoos, 2000; Kevan, 2003). Thus, the emotional and behavioral disturbances that are common in persons with CDB (Van Dijk and de Kort, 2005; Jacobsen et al., 2009; Nelson et al., 2013) may be attributable to limited communicational abilities. Accordingly, providing support and guidance to parents and their children to improve their reciprocal communication and emotional connection to each other may result in preventing the development of emotional and behavioral problems in children with CDB (Martens et al., 2014a,b). Emotional connection and a mutually satisfying relationship create emotional availability. The concept of emotional availability refers to emotional openness and reciprocity to each other’s signals in parent-child relations (Biringen and Easterbrooks, 2012). Emotional availability consists of the adult’s sensitivity, non-hostility, non-intrusiveness, and ability to structure play with the child (Biringen, 2008). Moreover, child responsiveness and involvement are part of emotional availability.

Emotional availability between infants and parents may be supported through imitation (Zeedyk et al., 2009). Imitation by a communication partner is a good interventional strategy, as it gives children with CDB the experience of being someone who can lead interaction and affect the behavior of others. This can radically change their role in interaction. Imitation also leads to turn-taking sequences with active participation by both partners of the dyad (Thelen et al., 1975; Kokkinaki and Kugiumutzakis, 2000; Hart, 2006). After being imitated, people with limited language skills have shown positive emotions (Caldwell, 2006; Hart, 2006; O’Neill and Zeedyk, 2006) and improved eye contact, bodily orientation, and partner proximity (Zeedyk et al., 2009; Contaldo et al., 2016).

To date, there are no studies exploring imitation and emotional availability in interactions between parents and children with CDB. This case report bridges the gap in research by examining imitative exchanges and emotional availability between a child with CDB and her mother before and after tactile imitation guidance. As imitation is characteristic in early intersubjective exchanges between typically developing infants and their parents (Trevarthen, 1979), the aim was to foster intersubjectivity in communication between a child with CDB and her mother through tactile imitation guidance. We aimed to investigate whether tactile imitation guidance would alter the frequency, length, and modality of imitation between a mother and her child with CDB. We were also interested in finding out whether tactile imitation guidance would improve emotional availability in the dyad.

## Methods

### Research Subjects

The research subjects were a mother and her 3-year-old daughter with Trisomy 13. The inclusion criteria for the child was dual sensory impairment and an early phase of language development (less than 10 spontaneous words or signs in the expressive vocabulary). The family was contacted through a contact person in the Finnish Federation of the Hard of Hearing (FFHH). An information letter regarding the study was sent to the family. After reading the information letter, the mother contacted the first author by telephone. During the call she was given the possibility to ask additional questions related to the study. The parents gave their informed written consent by mail. Based on the information given by the mother, the mother was 31 years old, employed, and with good physical and mental health. Thus, there were no maternal risk factors for dysfunction in the parent-child interaction. Ethical approval and a research permit were obtained from the Hospital District of Helsinki and Uusimaa.

The child is referred to as Emma in this paper. Emma had congenital visual and hearing impairments and she was almost blind. Her functional use of vision was restricted to perception of some light and black and white stripes. Emma was found to react to speech with hearing aids at 60/65 dB and music at 55 dB and had at least a moderately severe hearing impairment. She had a severe learning disability, a congenital heart defect, epilepsy, and severe problems in her motor development, which may have all challenged her in developing tactile abilities. Emma could not stand or walk independently and needed support when seated. She was able to use her hands, especially her fingers, in exploring objects that were brought near to her. The family had received communication support from a local center for persons with developmental disabilities. A communication adviser from the center had been seeing Emma weekly, mainly in the kindergarten. Emma’s family had also participated in a multidisciplinary individual rehabilitation course in the FFHH. Emma’s parents had been taught some manual signs, but no tactile signs. Emma had one sign, *to drink*, in her spontaneous use. She made the sign in the air. Her ability to comprehend speech and signs was unclear. The parents used tactile modality in making contact with Emma and interacted with her mainly vocally. Moreover, the parents used some objects of reference with Emma for anticipating daily routines. Emma expressed herself through vocalization, smiling, and bodily means.

### The Data

The data consist of videotaped play sessions between Emma and her mother. The researcher met the family 10 times and play sessions were videotaped during nine visits (I–II and IV–X). Session III was used to introduce the use of imitation to the mother. Sessions IV to X included imitation guidance. Recordings were made twice per week and on two consecutive days. Sessions from I to X were recorded over a 6-week period. The long time for data collection allowed the mother to practice imitation between sessions III and X. Each recording lasted from 8–10 min. Four sessions (I,IV,VIII, and X) between Emma and her mother were analyzed. These four recordings were chosen for analysis because they had the same participants (Emma and her mother), the same location for recordings (home), and the same interactional context (face-to-face interaction). The recordings from sessions II, V, VI, VII, and IX were left out of the analysis due to them having different participants (father in sessions II and V), Emma’s sleepiness due to a different location for recordings (sessions VI and VII), and technical reasons (session IX). The process of data collection is presented below. The bolded numbers refer to the sessions analyzed.


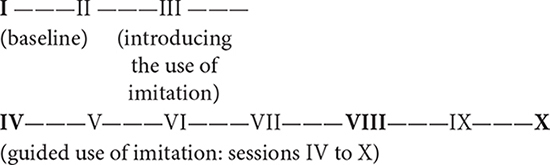


### Procedure for Tactile Imitation Guidance

In session I, the mother was asked to play with Emma as she normally would. Accordingly, play with and without toys was recorded. As Emma more actively participated in the play without toys, the first author together with the parents chose it for context for tactile imitation guidance. Based on the baseline observations, it was noticed that the mother often used vocal imitations. She also imitated gestures such as head-shaking, but due to her sensory impairments Emma was unable to perceive these imitations. In session III, as well as in guided sessions IV–X, the mother was given information about the use of imitation in interaction (e.g., reasons why imitation is used in interventions with individuals with limited language skills). Besides vocalization the mother was encouraged to imitate gestural expressions in a way that imitations were perceivable for Emma through tactile modality. For instance, the mother could make her imitation of a “shaking head” gesture tactile by guiding Emma to hold her hand on her mother’s face during imitation. Alternatively, she could make the imitative movement on Emma’s body, using the hand-under-hand strategy (Chen and Downing, 2006) or bodily contact for transmitting the information related to imitations. If Emma continued the imitative exchange, the mother was encouraged further to respond to her imitatively in order to create “imitative dialogues.” Nonetheless, the aim was to keep the interaction between Emma and her mother as natural as possible and use tactile modality only to enrich the interaction. During sessions III–X, the first author and the mother watched and discussed video clips of play sessions between Emma and her mother, and video clips related to imitation. They also practiced tactile imitation together with Emma during the play. The guidance was performed by the first author.

### Data Analysis

The five most active minutes in terms of Emma’s interaction from each recording were selected for the imitation analysis (c.f. Sanefuji et al., 2009). The verbal and non-verbal expressions of Emma and her mother were transcribed using Windows Media Player software. The analysis of emotional availability was based on full recordings.

### Frequency, Length, and Communication Modes Used in Imitations

A coding system devised by O’Neill and Zeedyk (2006) with modifications was used to analyze imitations.

*Imitative bouts* were identified when Emma or her mother were imitating the vocalizations, gestures, or actions of the other. An imitative bout included at least an expression and its imitation. The initiator of the imitative bout was marked. In order for an imitative bout to be recognized, the imitative response of Emma or her mother had to appear within 3 s of the end of the initial expression. The same time limit was used for Emma and her mother to keep the coding system consistent. A longer time than in O’Neill and Zeedyk (2006) was used, because 3 s were often needed for Emma’s imitative response to appear. The length of an imitative bout was calculated by identifying its complete *number of rounds*. If an expression was imitated only once and no more imitative turns were detected, the imitative bout had a length of one round. If similar expressions were produced during several turns, an imitative bout had several rounds.

Lastly, the *communicative mode* of each imitative bout was marked. In the original system by O’Neill and Zeedyk (2006) two categories were used: vocal (words and non-words) and physical imitation (gestures, actions, and manual signing). The categories were modified for the present study: (1) *vocal imitations* (e.g., vocalizations, imitative sighs, and whispers); (2) *gestural imitations* (e.g., head-shaking); (3) *tactile imitations* (e.g., the mother touched Emma in similar way as she had touched her); (4) *gestural-vocal imitations* (e.g., simultaneous vocalization and shaking head gesture); (5) *vocal-tactile imitations* (e.g., the mother imitated Emma vocally and traced the movement of her mouth onto Emma’s hand). The category of gestural-tactile imitations was not applied because the mother did not use this modality in her imitations. The coding of the communicative mode of an imitative bout was made in the following way: If the mother imitated Emma’s “ah” sound by saying “ah,” vocal imitation was marked. Similarly, if Emma shook her head and the mother imitated it by shaking her head, a gestural imitation was coded.

### Emotional Availability Scales

Emotional Availability Scales (EAS; 4th ed.) is an observational method that evaluates the quality of parent-child interaction (Biringen, 2008). EAS have four subscales for adults (sensitivity, structuring, non-hostility, and non-intrusiveness) and two for children (responsiveness and involvement). All the scales range from 1 to 7 (1 = non-optimal, 2.5/3 = somewhat [insensitive], 4 = inconsistent, 5.5/6 = moderate, 7 = optimal). *Sensitivity* refers to the ability of the adult to be warm and emotionally connected to the child, whereas *structuring* is reflected in a parent’s ability to structure the play with the child successfully. *Non-hostility* refers to the adult’s behavior that is free of negativity and *non-intrusiveness* relates to the adult’s capacity to be available to the child without intervening in his or her autonomy. *Child responsiveness* indicates how much the child is emotionally responsive to the adult, whereas *child involvement* is reflected in the degree to which the child addresses his or her initiatives to the adult. EAS is a global judgment of the recordings and does not use counts of discrete behaviors. When using EAS the coder evaluates, for instance, if the adult is able to read the child’s emotional signaling or if she/he is only behaviorally doing the right things. The EAS guidelines for children with disabilities were followed in rating (e.g., the coder has to keep in mind the disabilities of the child and their implications for behavior in the context; Biringen, 2008).

### Reliability

The reliability of the coding procedure related to communication modes used in imitative bouts was measured using intra-rater and inter-rater reliability tests. Intra-rater tests were made for all data by the first author. The agreement was 95%. The inter-rater reliability test was made by an independent second coder who had experience in working with children with multiple disabilities and who was fluent in Finnish Sign Language. The first author trained the second coder in using the imitation coding system. The training was carried out with the data that was not used for analyzing reliability. The second coder evaluated 25% of the data and the inter-rater agreement was 88%. The first author, who is trained in EAS, rated the videotapes with EAS. The accuracy and consistency of the EAS ratings were established by using another second coder who is a method trainer in EAS. The first and second coders negotiated the rating for one recording (25% of the data), and a reliability check was made for two recordings (50% of the data). The inter-rater agreement was 100% with a 1-point difference between the coders. Thus, both coders rated the quality of interaction into the same category in all the dimensions.

## Results

### Behavioral Changes During Physical Interaction

**FIGURE 1:**
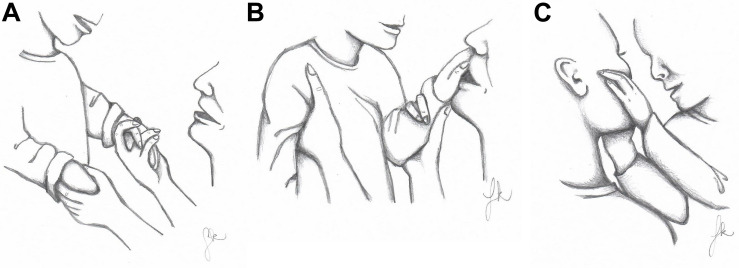
**(A–C)** Physical interaction and communication modes used in imitations before and after tactile imitation guidance. Drawings by Saara Koivula.

During all sessions, the mother interacted with Emma by playing with her face-to-face without using toys. Before imitation guidance was given in play session I, the mother physically held Emma either by her hands (Figure 1A) or under her armpits (Figure 1B). After tactile imitation guidance was provided in play sessions IV, VIII, and X, the mother was able to hold Emma mainly under her armpits. This allowed Emma to constantly move her hands freely to touch her mother. Emma did not make the gesture of placing her hands on her mother’s face before guidance in play session I. After the guidance, she once placed her hands on her mother’s face in session IV and this gesture appeared increasingly in session VIII. In session X, Emma placed her hands on her mother’s face more frequently, also beyond the imitative bouts (Figure 1C). Similarly, Emma’s positive emotional expressions appeared infrequently connected to her mother’s imitation in play session I. By contrast, in the sessions with guidance, Emma’s positive emotional expressions increased notably.

### Frequency and Length of Imitative Bouts

The frequency and length of the imitative bouts are shown in Table 1. Most of the bouts were initiated by the mother in all sessions. Play session I before tactile imitation guidance included 20 short imitative bouts, whereas after the guidance in session X the bouts lasted longer. Thus, after the guidance the length of the imitative bouts increased. In each session, Emma initiated 1–2 imitative bouts and the length of the imitative bouts initiated by her varied between 1 1/2 and 7 1/2 rounds.

**TABLE 1:** Frequency and length of imitative bouts initiated by Emma and her mother.

	Mother	Emma
**Baseline**		
Session I	16 (1 Round)	1 (1 1/2 Rounds)
	3 (2 Rounds)	
**Sessions with tactile imitation guidance**		
Session IV	4 (1 Round)	1 (7 1/2 Rounds)
Session VIII	11 (1 Round)	1 (1 1/2 Rounds)
	2 (2 Rounds)	
Session X	10 (1 Round)	1 (1 1/2 Rounds)
	3 (2 Rounds)	1 (2 1/2 Rounds)
	1 (3 Rounds)	
	1 (4 Rounds)	
	1 (9 Rounds)	
	1 (11 Rounds)	

**The length of a bout is marked in parentheses.**

### Communication Modes Used in Imitations

The communication modes used in the imitations by Emma and her mother in this study are presented in Table 2. Before guidance in play session I, most of the mother’s imitations were vocal. Tactile modality was used only in connection with one vocal imitation. In this vocal-tactile imitation, the mother imitated Emma’s laughter by shaking Emma’s hands in the same rhythm as her laughter. The mother’s imitations became more tactile in the sessions with guidance. In session IV the mother made one tactile imitation by touching Emma’s arm in the same way Emma had touched her. In vocal-tactile imitations the mother imitated Emma’s vocalization by tracing the movement of her mouth onto Emma’s hand (Figure 1B). In session VIII the mother’s vocal-tactile imitations increased to 10. In these imitations, the mother touched Emma’s head with her nose, forehead, or head while speaking or vocalizing, kissed Emma’s cheek, or imitated Emma’s vocalization by tracing the movement of her mouth onto Emma’s hand. In session X the mother did not use tactility in her imitations as frequently as in session VIII because Emma spontaneously touched her mother’s face before the mother imitated Emma’s vocalizations. Thus, there was no need to add tactility in imitations as Emma continuously explored her mother’s face during and beyond her mother’s imitations.

**TABLE 2:** Communication modes used in imitations by Emma and her mother.

	Baseline	Sessions with tactile imitation guidance		
		
	Session I	Session IV	Session VIII	Session X
Vocal	10*(E 1)*	0*(E 1)*	2	12*(E 1)*
Gestural	5	0	1*(E 1)*	0
Tactile	0	1	0	0
Gestural-vocal	3	1	0	2*(E 1)*
Vocal-tactile	1	2	10	3
All imitative responses including tactile modality in interaction^*a*^	1	3	12	10

**Emma’s imitative expressions are marked with E and written in italics in parentheses. *^*a*^This category includes both the turns in which the mother used tactility in imitation and the turns when Emma was exploring her mother’s mouth or face during her mother’s imitation.***

Emma made few imitations and her imitative responses were single utterances belonging to her vocal or gestural repertoire (see Table 2). Sometimes her mother made several repetitions of an utterance before Emma imitated it. Emma’s gestural imitative responses were imitations of her mother’s gestures that had included tactility.

### Emotional Availability of Emma and Her Mother

The results for the emotional availability of Emma and her mother are presented in Table 3. Before tactile imitation guidance in play session I, the mother’s score for sensitivity, structuring, and non-intrusiveness was 5 for all the dimensions, whereas in guided sessions IV, VIII, and X her scores ranged between 6 and 7. Likewise, in play session I, Emma’s scores for responsiveness and involvement ranged between 3 and 4.5. In guided play sessions IV, VIII, and X, her scores were higher, ranging between 3.5 and 6. The most notable change was observed in Emma’s increased involvement in the interaction. In sessions VIII and X Emma vocalized and tactilely explored her mother’s face more frequently than in the previous sessions.

**TABLE 3:** Emotional availability scores of Emma and her mother in play sessions.

	Baseline	Sessions with tactile imitation guidance		
		
	Session I	Session IV	Session VIII	Session X
**Emotional availability scales: mother**				
Sensitivity	5	6	7	7
Structuring	5	6	7	7
Non-intrusiveness	5	6	7	7
Non-hostility	7	7	7	7
**Emotional availability scales: Emma**				
Child responsiveness	4.5	5	6	6
Child involvement	3	3.5	6	5.5

**All the scales range from 1 (low) to 7 (high).**

## Discussion

The results of this study show that the mother was responsible for taking an active role in the imitations compared to Emma. This finding that most imitative bouts were initiated by the adult carers is consistent with the results of O’Neill and Zeedyk (2006). Our results clearly demonstrate that after tactile imitation guidance was provided the imitative exchanges between Emma and her mother lasted longer, especially in the last session, which appears to be related to the fact that through the tactile modality, Emma was more able to perceive her mother’s imitations. Furthermore, the mother’s imitations during the interaction became more tactile in the sessions when guidance was given. Similarly, Martens et al. (2014a) found that communication partners can be trained in tactile emotion sharing with persons with CDB. When practicing the use of tactile modality in interaction, parents take on a learner’s role instead of communicating in such a way that is automatic and effortless. This learning process changes the interaction with children with CDB to be more equal, and not only gives parents new skills but also sensitizes them in perceiving the subtle gestures and touches of the child that are often connected to tactile experiences (Hart, 2010).

Regarding the EAS, the results indicate an increase in the sessions with tactile imitation guidance compared to baseline observations. For instance, the results of the raw scores showed that the mother scored higher in the sensitivity, structuring, and non-intrusiveness scales, whilst Emma scored higher in the responsiveness and involvement scales when tactile imitation guidance was provided during play sessions IV, VIII, and X (see Table 3). Although the EAS scores of the mother were elevated before guidance was given, the EAS scores of Emma clearly improved after guidance was provided. For instance, the scores of the responsiveness and involvement scales improved from a risk zone to a non-risk zone within the EAS framework. This positive link between touch and emotional availability is in line with earlier findings of emotional availability and touch in children who are deaf or hard-of-hearing (Pipp-Siegel et al., 1999; Paradis and Koester, 2015). EAS seems an appropriate tool for capturing the emotional quality of interactions between children with CDB and their carers as the coding system is flexible (Biringen et al., 2005; Biringen, 2008). However, it may be difficult for an EAS-coder to assess the quality of interaction between children with CDB and their carers without expertise in CDB, especially in tactile communication. More studies are needed to validate the applicability of EAS in assessing the emotional quality of interactions between children with CDB and their carers.

### Implications for Practice and Further Research

The results of this study suggest that supporting imitation between parent and child especially through the tactile modality might be an appropriate interventional strategy to support the interaction, if the child with CDB is in the early stages of language development, takes fewer communicative initiatives, or is hard to make contact with (see also Contaldo et al., 2016). However, imitation is not the only strategy for supporting interaction in children with CDB or similar conditions, nor is it sufficient to promote communication and language development alone. Neither should imitation be seen as an aim of interaction, but rather as a starting point in a dialogue (cf. Linell, 2009). Nonetheless, imitation might be a powerful means for supporting interaction in the early phases of language development when many other interventional strategies might not be suitable yet. However, further studies are needed to confirm the usefulness of tactile imitation guidance when supporting interaction in a child with CDB. For instance, tactile imitative exchanges between parents and children with CDB may have the same importance for the development of their tactile language skills (e.g., tactile signs) that vocal imitative exchanges between parents and their non-CDB children have for the development of speech (see Papoušek and Papoušek, 1989).

## Limitations

There are limitations in the present study that need to be considered. First, defining the imitator was not always clear. For instance, Emma’s response latencies could sometimes be longer than 3 s, which meant her delayed responses were not coded as imitations. The coding system, however, gave a clear structure for the analysis, and it made it possible to capture most of the dynamics related to imitation. Second, a longer baseline would have been needed to get a more comprehensive picture of the change that tactile imitation guidance made to the interaction between Emma and her mother. Third, a follow-up period would have been needed to provide information about lasting effects. Last, the case study design limits the generalizability of the findings.

## Conclusion

The most important finding of the present study was that before tactile imitation guidance the mother imitated Emma mainly vocally, whereas the use of tactility in imitations notably increased after the guidance. Both Emma and her mother scored higher on emotional availability in sessions in which the mother used tactility in imitations. The results suggest that the mother’s use of imitation with tactility improved the quality of the interaction. Further investigation is required to discover the best strategies for guidance for parents with children with CDB who are at the early stages of language development. A good quality of interaction between parents and children with CDB forms the best prerequisite for the children’s development and promotes general well-being in families.

## Data Availability Statement

The data cannot be shared for ethical restrictions.

## Ethics Statement

The studies involving human participants were reviewed and approved by the Helsingin ja Uudenmaan Sairaanhoitopiiri, Eettinen Toimikunta IV, THL, Biomedicum 1, PL 104, 00215 Helsinki, Finland. Written informed consent to participate in this study was provided by the participants’ legal guardian/next of kin.

## Author Contributions

SP, MD, SS, and ML contributed to the design of the study. SP collected the data and wrote the first draft of the manuscript. SP and SS analyzed the data. All authors contributed to manuscript revision and read and approved the submitted version.

## Conflict of Interest

The authors declare that the research was conducted in the absence of any commercial or financial relationships that could be construed as a potential conflict of interest.
